# A Randomized Cadaver Study Comparing First-Attempt Success Between Tibial and Humeral Intraosseous Insertions Using NIO Device by Paramedics: A Preliminary Investigation

**DOI:** 10.1097/MD.0000000000003724

**Published:** 2016-05-20

**Authors:** Lukasz Szarpak, Zenon Truszewski, Jacek Smereka, Paweł Krajewski, Marcin Fudalej, Piotr Adamczyk, Lukasz Czyzewski

**Affiliations:** From the Department of Emergency Medicine (LS, ZT), Medical University of Warsaw, Warsaw; Department of Emergency Medical Service (JS), Wroclaw Medical University, Wroclaw; Department of Forensic Medicine (PK, MF); Student Research Circle at the Department of Emergency Medicine (PA); and Department of Nephrologic Nursing (LC), Medical University of Warsaw, Warsaw, Poland.

## Abstract

Medical personnel may encounter difficulties in obtaining intravenous (IV) access during cardiac arrest. The 2015 American Heart Association guidelines and the 2015 European Resuscitation Council guidelines for cardiopulmonary resuscitation (CPR) suggest that rescuers establish intraosseous (IO) access if an IV line is not easily obtainable.

The aim of the study was to compare the success rates of the IO proximal tibia and proximal humerus head access performed by paramedics using the New Intraosseous access device (NIO; Persys Medical, Houston, TX, USA) in an adult cadaver model during simulated CPR.

In an interventional, randomized, crossover, single-center cadaver study, a semi-automatic spring-load driven NIO access device was investigated. In total, 84 paramedics with less than 5-year experience in Emergency Medical Service participated in the study. The trial was performed on 42 adult cadavers. In each cadaver, 2 IO accesses to the humerus head, and 2 IO accesses to the proximal tibia were obtained.

The success rate of the first IO attempt was 89.3% (75/84) for tibial access, and 73.8% (62/84) for humeral access (*P* = 0.017). The procedure times were significantly faster for tibial access [16.8 (interquartile range, IQR, 15.1–19.9] s] than humeral access [26.7 (IQR, 22.1–30.9) s] (*P* < 0.001).

Tibial IO access is easier and faster to put in place than humeral IO access. Humeral IO access can be an alternative method to tibial IO access.

Trial Registration: clinicaltrials.gov Identifier: NCT02700867.

## INTRODUCTION

In the out-of-hospital emergency settings, rapid vascular access is often required in order to administer drugs and fluids in critical patients, and is particularly important during cardiopulmonary resuscitation (CPR). Medical personnel may encounter difficulties in obtaining intravenous (IV) access during cardiac arrest; the heart does not work as a pump, which causes veins to collapse. The average time needed for peripheral IV catheterization is reported to be between 2.5 and 16 minutes in patients with difficult IV access.^1,2^ The 2015 American Heart Association (AHA) guidelines and the 2015 European Resuscitation Council (ERC) guidelines for CPR suggest that rescuers establish intraosseous (IO) access if an IV line is not easily obtainable.^3,4^

The IO access is usually established in the proximal part of the tibia (near the tibial tuberosity), or in its distal part (near the medial ankle). Other penetration sites used for injection include the humeral bone head, radial bone, or femoral bone. The IO access allows the patient to be treated immediately by facilitating the administration of fluids and medications.^5^ All drugs and IV solutions may be administered through IO access^6,7^; however, it should not last longer than 24 hours and should be discontinued as soon as peripheral or central IV access has been established. The most frequent complications include hematoma, inflammation, and bone fractures.^8^

The aim of the study was to compare the success rates of the IO proximal tibia and proximal humerus head access performed by paramedics using the New Intraosseous access device (NIO; Persys Medical, Houston, TX, USA) in an adult cadaver model during simulated CPR.

## METHODS

### Study Design

The study was approved by the Bioethics Committee of the Medical University of Warsaw, Poland (approval No.: KB/22/2015), and registered in the ClinicalTrials database (www.clinicaltrials.gov, NCT02700867). It was an interventional, randomized, crossover, single-center study, carried out between February and March 2016. All the procedures took place at the Medical University of Warsaw, Poland, laboratory of the Department of Forensic Medicine.

Power analysis was performed, which revealed that a sample size of 40 per group would provide 80% power to detect a moderate effect size difference of 1.0 (or approximately 1.0) standard deviation (SD) between the means at the alpha level of 0.05 (Statistica Software, version 12.5; StatSoft, Inc., Tulsa, OK).

In total, 84 paramedics with less than 5-year experience in Emergency Medical Service (EMS) participated in the study. The participants had not been trained on either of the IO access devices before the study began. Persons with previous experience in IO access were excluded from the study. All participants were verbally informed about the intention of the study and they provided their written informed consent to take part in the trial.

### Cadaver Subjects

Eligible fresh cadavers aged 18 to 65 years at the time of death were screened for potential inclusion in collaboration with the Department of Forensic Medicine, Warsaw Medical University, Warsaw, Poland. Fresh cadavers were defined as cadavers up to 72 hours after death. Among the exclusion criteria, there were the targeted bone fracture, bone tumor or myeloproliferative malignancy, previous orthopedic procedures near the insertion site, prosthetic limb or joint, IO access obtained recently, inability to locate landmarks because of excessive tissue, or infection at the insertion site. During the study period, 42 fresh cadavers were identified. Two IO accesses to the humerus head and 2 IO accesses to the proximal tibia were obtained for each cadaver body.

### Procedures

**FIGURE 1:**
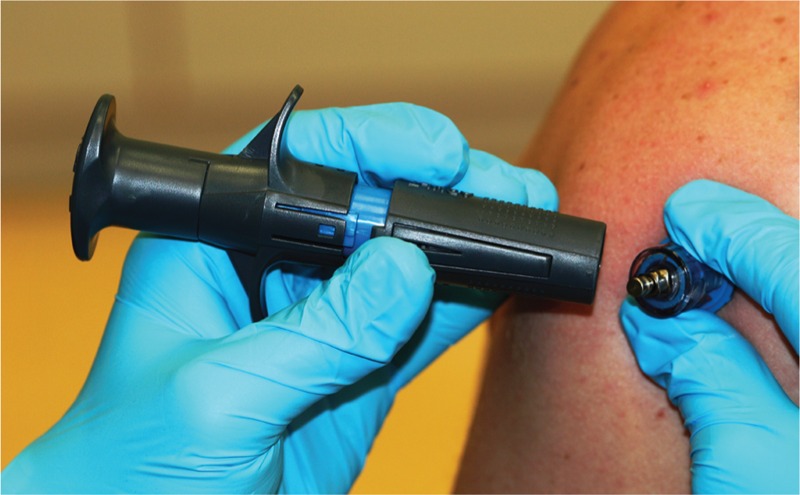
The NIO Intraosseous adult access device.

A disposable, spring-load driven NIO access device with an integrated trigger—a semi-automatic IO access device approved by the Food and Drug Administration—was investigated. It contains a 15-gauge, 35-mm stainless cannula with a constant insertion depth for adults and integrated needle stabilizer; therefore, it can be used on the tibia or humerus (Figure 1).

Before the study, all participants received 5-minute in-service presentation regarding the method of establishing an IO access with the use of the NIO. Then, they performed 3 insertions with the NIO Training Kit to make sure they were familiar with the technique.

**FIGURE 2:**
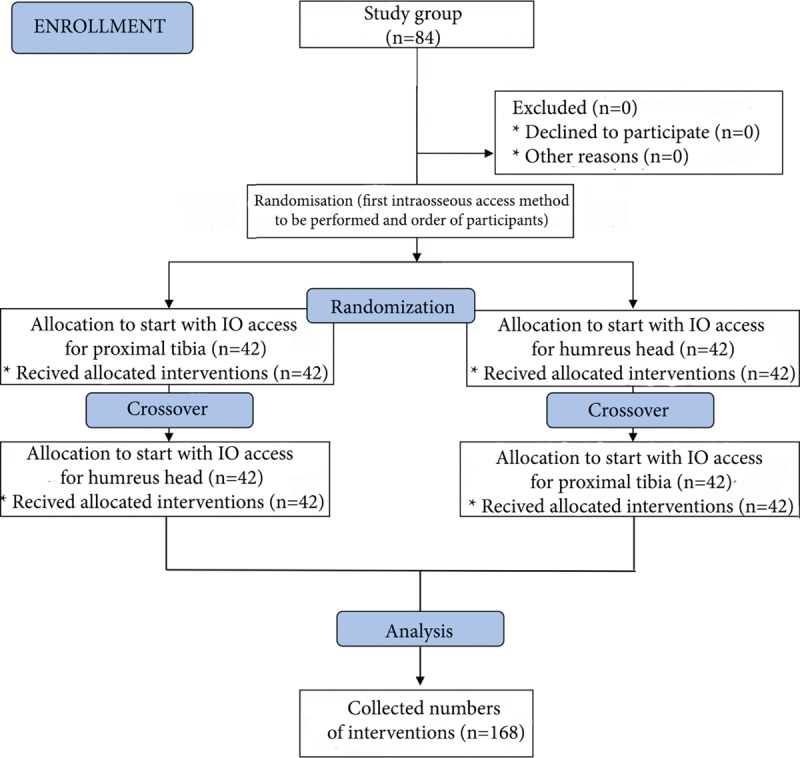
A flow chart presenting the study design and participants recruitment according to CONSORT statement.

**FIGURE 3:**
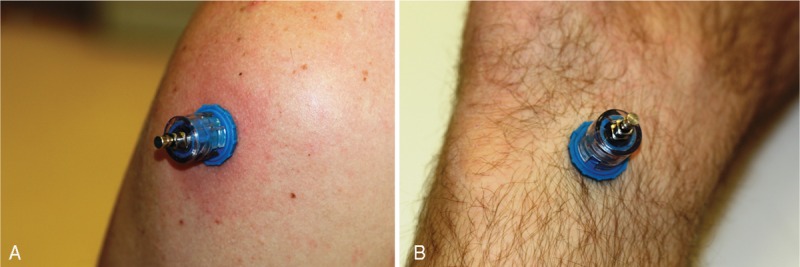
Intraosseous access founded by the NIO device: (A) into proximal tibia; (B) into humerous head.

A research randomizer program was used (www.researchrandomizer.org) to divide the participants into 2 groups and to determine the order in which the participants would perform the IO access and the order of the anatomic sites (humeral head or proximal tibia) of performing the IO access (Figure 2). The first group attempted to perform the IO access in the tibia, and the second in the humerus (Figure 3). After completing each IO procedure, participants had a 5-minute break before performing IO access using NIO in the next location. The participants were allowed only 1 IO access attempt with each location. Each of them performed an IO cannulation in the humeral head and proximal tibia. They were reminded before each attempt that the “patient” needed emergency IV access as quickly as possible; this was to provide the feeling of time pressure that would be present in real emergency situations. Chest compression was performed with the use of Lifeline ARM chest compression system (ARM; Defibtech, Guilford).

### Outcome Measures and Data Collection

The main outcome was the time of IO cannulation. It was measured in seconds, and defined as the time interval between taking the IO device out of the original packaging to the actual insertion of the needle into the bone and problem-free IO administration of 10 mL saline solution as a test dose directly through the needle. The secondary outcome was the success rate of IO cannulation on the first attempt. An insertion was labeled successful if it had a stable position on the bone and allowed the infusion of fluid (10 mL) without extravasation. Failure was defined as extravasation or unsuccessful (first) effort of IO insertion. In addition, the IO insertion was confirmed by ultrasonography with the application of a linear 6 MHz probe. Furthermore, complications of the IO cannulation were recorded.

After the procedure completion, each participant filled a questionnaire in which they subjectively rated the ease of NIO use (1–5; 1 = very difficult, 5 = very easy), the ease of NIO use versus a peripheral IV line (easier, the same, harder), the speed versus a peripheral IV line (slower, the same, faster), as well as the willingness to use the device in future sudden cardiac arrest (SCA) scenarios (no, maybe, yes).

### Statistical Analysis

All study data were entered into an electronic database (Microsoft Excel 2010; Poland) (Microsoft Corp, Redmond, WA, USA) and evaluated with the use of Statistica Package Software, version 12.5.

The results were presented as absolute values, percentages, medians and interquartile ranges (IQRs), or means and SDs. The Kolmogorov–Smirnov test was applied to check for normal distribution. As this was a randomized crossover trial, pairing was taken into account in the statistical analysis. McNemar test was used for comparing the cannulation success rates of the humeral head and proximal tibia. The 2-sided Wilcoxon signed-rank test allowed to compare the procedure time. The participants’ subjective opinions were compared with the use of the Stuart–Maxwell test. The value of *P* < 0.05 was considered statistically significant.

## RESULTS

The study was carried out between February and March 2016. In total, 84 paramedics (27 women and 57 men aged 27.6 ± 4.2 years) participated in the study.

The success rate of the first IO attempt was 89.3% (75/84) for tibial access and 73.8% (62/84) for humeral access (*P* = 0.017). All unsuccessful attempts to the tibia IO access were bound with a poor relief angle between the device and the bone, and with inserting the needle at the wrong angle. The reason for all unsuccessful attempts to the humerus IO access was an incorrect locating of the humeral head.

**FIGURE 4:**
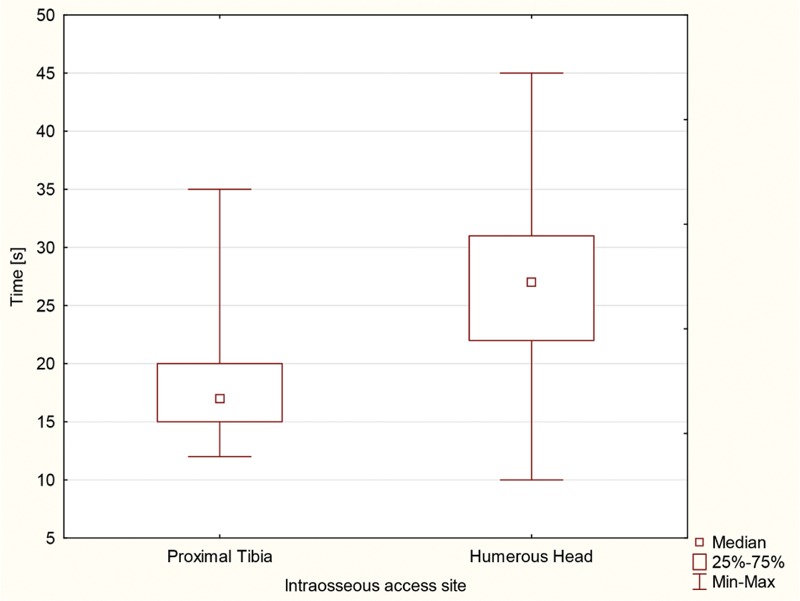
The time needed to perform the IO access.

The procedure times were significantly faster for tibial access [16.8 (IQR, 15.1–19.9) s] than humeral access [26.7 (22.1–30.9) s] (*P* < 0.001). The time needed to perform the procedure is presented in Figure 4.

The participants assessed the IO access into proximal tibia as easier to obtain than the IO access into proximal humerus (*P* < 0.001; Table 1). As many as 81 of the 84 (96.4%) participants stated that they would use the NIO device in a future SCA scenario; 3 participants were hesitant. All participants (100%) perceived the IO access with the use of the NIO device as faster in their hands than placing a peripheral IV line, and all of them rated the NIO device easier than inserting a peripheral IV line, which applies to the IO access both in proximal tibia and in proximal humerus.

**TABLE 1:**
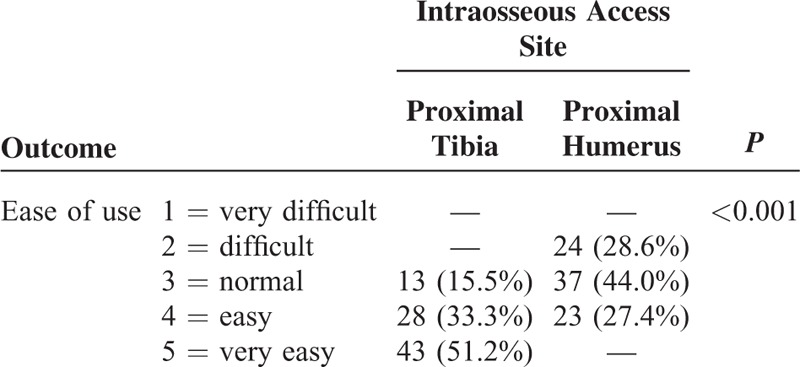
Comparison of the Ease of Using the New Intraosseous Access Device for the Proximal Humerus and Proximal Tibia Access

## DISCUSSION

In the trial described, the authors compared the necessary procedure times and success rates of the first attempt to obtain IO access in proximal tibia and humerus head in adult cadavers under simulated resuscitation. To our knowledge, this was the first randomized crossover trial to assess the frequency of the first-attempt success between humeral and tibial IO access. There are no randomized crossover trials presented in literature that would compare the tibial and humeral IO access obtained with the NIO device. Results demonstrated that the tibial IO route was the most effective method of gaining vascular access during simulated CPR.

The IO access was first described in 1922^9^ and was used in a systematic manner during World War II.^10^ Over the 9 decades, it has been applied as a safe alternative to peripheral venous access (PVA). According to the ERC and AHA guidelines for CPR, since 2010, the IO access has become a standard of care in adult advanced life support, and the first recommended alternative PVA in adult SCA patients.^3,4^ Moreover, according to the ERC and AHA guidelines, the IO access is the first recommended vascular access in pediatric emergencies such as SCA.^11,12^

Rapid intravascular access is an essential component of CPR, especially in nondefibrillation rhythms, allowing administration of epinephrine or other drugs or fluids. However, in emergency patients, PVA might be difficult or impossible to obtain, especially in dehydrated patients, those in hypovolemic shock, obese, IV drug users, following chemotherapy, or under SCA. As reported in many studies, failure rates of PVA in emergency conditions equal around 10% to 40%.^1,12,13^ Of course, there are many alternative vascular access techniques under CPR with difficult PVA access, such as central venous cannulation (CVC) or ultrasound-guided PVA.

CVC results in shorter drug circulation times and higher peak drug concentrations than PVA. Moreover, CVC is relatively time-consuming and associated with numerous complications in the emergency setting, such as line malposition, hematoma, arterial puncture, venous thrombolysis, pneumothorax, or catheter-related infections. The complications are reported to affect around 15% to 20% of cases.^14–16^ Lee et al^17^ show that first-pass success was significantly higher for the IO access than for a central venous catheter (90.3% vs. 37.5%; *P* < 0.001). Eisen et al^16^ also indicated that landmark-guided CVC in femoral or subclavian veins was associated with first-attempt failure rates of up to 40%. The next mentioned alternative for standard PVA, ultrasound-guided IV access, enabled first-attempt success rates of 46% to 84%.^1,2,13^ In the present study, the first-pass IO success rate for the proximal tibia access site was 89.3%. The results obtained for proximal tibia IO access were similar to those of prior observational studies among paramedic, as well as emergency physicians.^17–20^ Wampler et al,^21^ in their study concerning the first-attempt success rate with humeral placement of the EZ-IO by paramedics among prehospital adult cardiac arrest patients, prove that the first-attempt successful placement referred to 91% of cases. Kurowski et al,^5^ in a study comparing BIG, EZ-IO, and Jamshidi devices, reported that the highest first-time attempt success rate, 91.59%, was observed with the BIG device, followed by the EZ-IO with 82.24% and the Jamshidi IO Needle with 47.66%. However, Brenner et al^22^ noted that an injection with the use of the EZ-IO was characterized by higher rates of efficacy and superior ease of use. Helm et al^23^ reported the overall success rate of EZ-IO as 99.6%, with the first attempt success rate of 85.9%. It is also worth pointing out that in the present study, the first-attempt success rate for humerus head IO access was 73.8%. The low efficiency was due to the wrong location on the humeral head—and the subsequent improper IO access. It should be emphasized that in many studies, the effectiveness of IO humeral head access proves lower than that of IO proximal tibia access. As shown in studies performed on pigs by Lairet et al,^24^ the humerus is a suitable alternative site for IO access, with a potential for higher flow rates than the proximal tibia when resuscitating a patient.

Academic research indicates that the average access time for the emergency scenario in PVA cases is 2 to 26.7 minutes.^25,26^ Landmark-guided CVC is a time-consuming procedure, with the average access time of 8 to 10.7 minutes,^16,17^ and in ultrasound-guided IV, the access time equals 6 to 20 minutes.^1,2,13^ Suyama et al^27^ show that the time to establish an IO infusion was significantly shorter than that for PVA infusion in a simulated adult emergency scenario. Moreover, Lee et al^17^ prove that mean placement times were significantly shorter for IO access than for CVC (1.2 vs. 10.7 min; *P* < 0.001). In a study by Hartholt et al,^28^ median insertion times ranged from 38 seconds for the Jamshidi 15G to 49 seconds for the BIG 15G and 62 seconds for the FAST1. Leidel et al^19^ demonstrated that the effectiveness of IO puncture was 80% with BIG and 90% with EZ-IO devices, whereas the respective results obtained by Sunde et al^29^ equaled 55% and 96%. Gerritse et al^30^ reported 71% and 73% with BIG for children and adults, respectively. Moreover, in the present study, the average IO access time was 16.8 seconds for the proximal tibia and 26.7 seconds for the humeral head. A longer IO access time in the event of the humeral head was also observed by Reades et al.^31^

Pharmacokinetic and pharmacodynamic studies have shown that the IO route is equivalent to an IV access for emergency drugs administration.^32,33^ Johnson et al^7,8^ observed that the IO delivered a higher concentration of epinephrine than the IV route at 30 seconds, which may constitute a survival advantage. Neufeld et al^32^ indicated that drugs and fluids infused through IO access quickly reached the central venous circulation at concentrations comparable with the CVC.

The technical problem indicated by other authors, difficulty in removing the stylet from the needle, was not observed in the present study.^33,34^ Draaisma et al^35^ demonstrated that the problem of the “stuck stylet” occurred in 3 of 40 cases. In the authors’ study, ineffective IO injections in the humeral head stemmed from an improper location of the injection site. It seems reasonable, therefore, to increase the emphasis on the correct puncture site location during the IO access training.

EMS personnel have to decide which alternative for IV access should be chosen. There are several possibilities for IO access to be performed in clinical practice. The simulated CPR settings for IO insertion are very similar to real practice settings even in cadavers there are fixated tissues and there is no circulation. The same dilemma and the same time for insertion should be spent in real practice and simulated cadaver CPR settings. In the authors’ opinion, the results can be directly translated into human studies and clinical practice.

## LIMITATIONS AND STRENGTHS

There are several potential limitations to the study. First, it was conducted on cadavers during simulated CPR. The cadavers were chosen intentionally, as according to the International Liaison Committee on Resuscitation, randomized clinical trials with cardiac arrest cases are unethical and cannot determine the expected CPR benefits.^36^ Moreover, the authors realize that studies on cadavers, in contrast to those among living subjects, are characterized by typical limitations, such as fixated tissues or no existing circulation. Bone marrow aspiration tests to verify the correct needle placement can be difficult or even impossible in cadavers; however, these difficulties can also be observed in living humans. To eliminate this limitation, only fresh cadavers were included in the study, and the ultrasound test was applied in order to finally confirm the IO location. Another limitation of the study is the fact that the research group constituted only of paramedics; they are, however, the ones in the EMS teams who most frequently face the urgent necessity of performing an intravascular access during CPR.

The strengths of the study include the randomized crossover procedure and the usage of a mechanical chest compression machine to simulate chest compressions.

## CONCLUSIONS

Tibial IO access is easier and faster to put in place than humeral IO access. Humeral IO access can be an alternative method to tibial IO access.
